# Perceived emotional expressions of composite faces

**DOI:** 10.1371/journal.pone.0230039

**Published:** 2020-03-10

**Authors:** Markku Kilpeläinen, Viljami Salmela

**Affiliations:** Department of Psychology and Logopedics, University of Helsinki, Helsinki, Finland; Bournemouth University, UNITED KINGDOM

## Abstract

The eye and mouth regions serve as the primary sources of facial information regarding an individual’s emotional state. The aim of this study was to provide a comprehensive assessment of the relative importance of those two information sources in the identification of different emotions. The stimuli were composite facial images, in which different expressions (Neutral, Anger, Disgust, Fear, Happiness, Contempt, and Surprise) were presented in the eyes and the mouth. Participants (21 women, 11 men, mean age 25 years) rated the expressions of 7 congruent and 42 incongruent composite faces by clicking on a point within the valence-arousal emotion space. Eye movements were also monitored. With most incongruent composite images, the perceived emotion corresponded to the expression of either the eye region or the mouth region or an average of those. The happy expression was different. Happy eyes often shifted the perceived emotion towards a slightly negative point in the valence-arousal space, not towards the location associated with a congruent happy expression. The eye-tracking data revealed significant effects of congruency, expressions and interaction on total dwell time. Our data indicate that whether a face that combines features from two emotional expressions leads to a percept based on only one of the expressions (categorical perception) or integration of the two expressions (dimensional perception), or something altogether different, strongly depends upon the expressions involved.

## Introduction

People are experts in the processing of the visual information provided by human faces [1,2]. We can quickly determine the identity, gender, approximate age and emotional state by looking at an individual’s face. When interpreting the emotional expression on a face, the eye and mouth areas represent the primary sources of information [3]. On one hand, eye contact is considered socially desirable and the ability to extract all necessary information while fixating in the eye region would be beneficial. On the other hand, the emotional expressions produced by the mouth region may carry more bottom-up saliency and, thus, may constitute a potentially more reliable source of information [4].

In general, research indicates that the relative roles of the eye region (i.e., the top half) and the mouth region (i.e., the bottom half) differ between emotional expressions. A study with partially masked faces (bubbles) found that individuals classified expressions as happy, surprised and disgusted primarily based on the mouth region, and anger primarily on the eyes [3]. Calder, Keane, Young and Dean [5] found that anger, fear and sadness were more quickly recognised from a stimulus containing only the top side of a face, happiness and disgust from the bottom side, and surprise equally well from both sides. Similar results have also been found in many experiments that have used composite expressions, i.e., stimuli where one expression in the top half of a face has been combined with another expression in the bottom half. For example, Calvo and Fernández-Martín [6] found that a happy non-fixated mouth biased participants’ perceptions of the expression presented in the fixated eye region more strongly than sad or angry mouth expressions. Finally, Calvo and Nummenmaa concluded that participants’ ability to make fast saccadic choices between happy and other faces was driven by the high salience of the happy mouth [4].

Studies that use composite expression stimuli have mainly examined how the processing speed or efficiency of one stimulus half (top or bottom) is affected by the other half. The general pattern suggests that a congruent expression leads to faster and more accurate processing whilst an incongruent expression renders processing slower and less accurate [7,8]. Much less attention has been devoted to the appearance of the various composite expressions. For example, does a happy mouth with angry eyes look happy, angry, something in between or something altogether different? To answer that question, the relative roles of the eyes and the mouth in dictating the perceived expression, should be determined. Traditionally, results of emotion research have been interpreted in the light of two distinct theories; the categorical theory proposed by Ekman and colleagues [9], and the dimensional theory proposed by Russell and colleagues [10].

The categorical theory suggest that we assign all emotions to a discrete set of core emotional categories. In contrast, the dimensional theory suggest that all our emotional experiences lie on continuums without sharp boundaries. Previously, it has been noted that different tasks might bias, but not fully determine [11], the subjects’ responses towards categorical perception (e.g., emotion identification) or towards dimensional perception (e.g., multi-dimensional scaling). Emotional processes are also affected by linguistics or verbalisation [12]; although we have a continuous experience of emotions, we have only a subset of terms/adjectives to describe them. In our setup, the categorical and the dimensional theories offer considerably different predictions. The categorical theory predicts that the perception is more or less the same as the perception of the expression conveyed by the dominant half of the face, whereas the dimensional theory suggests that the perceived expression is more likely to lie somewhere along a smooth perceptual continuum between two expressions [11].

Although the categorical and dimensional theories’ predictions clearly differ concerning our study, it must be pointed out that many researchers now consider these two theories to provide complementary tools for the understanding of human emotions [13]. Interestingly, a recent brain imaging study found some brain regions to respond to emotional facial expressions in a dimensional manner, some in a more categorical manner [14]. Furthermore, recent theories suggest a constant interplay between emotion and cognition so that we cannot study one without the effect of the other [13]. Thus it is possible that while we are evaluating facial expressions in terms of emotions, we also evaluate the mental state of the person we are looking at. Recent study has shown that a vast set of mental states can be expressed with faces and those can be perceived quite similarly across subjects [15].

**Fig 1:**
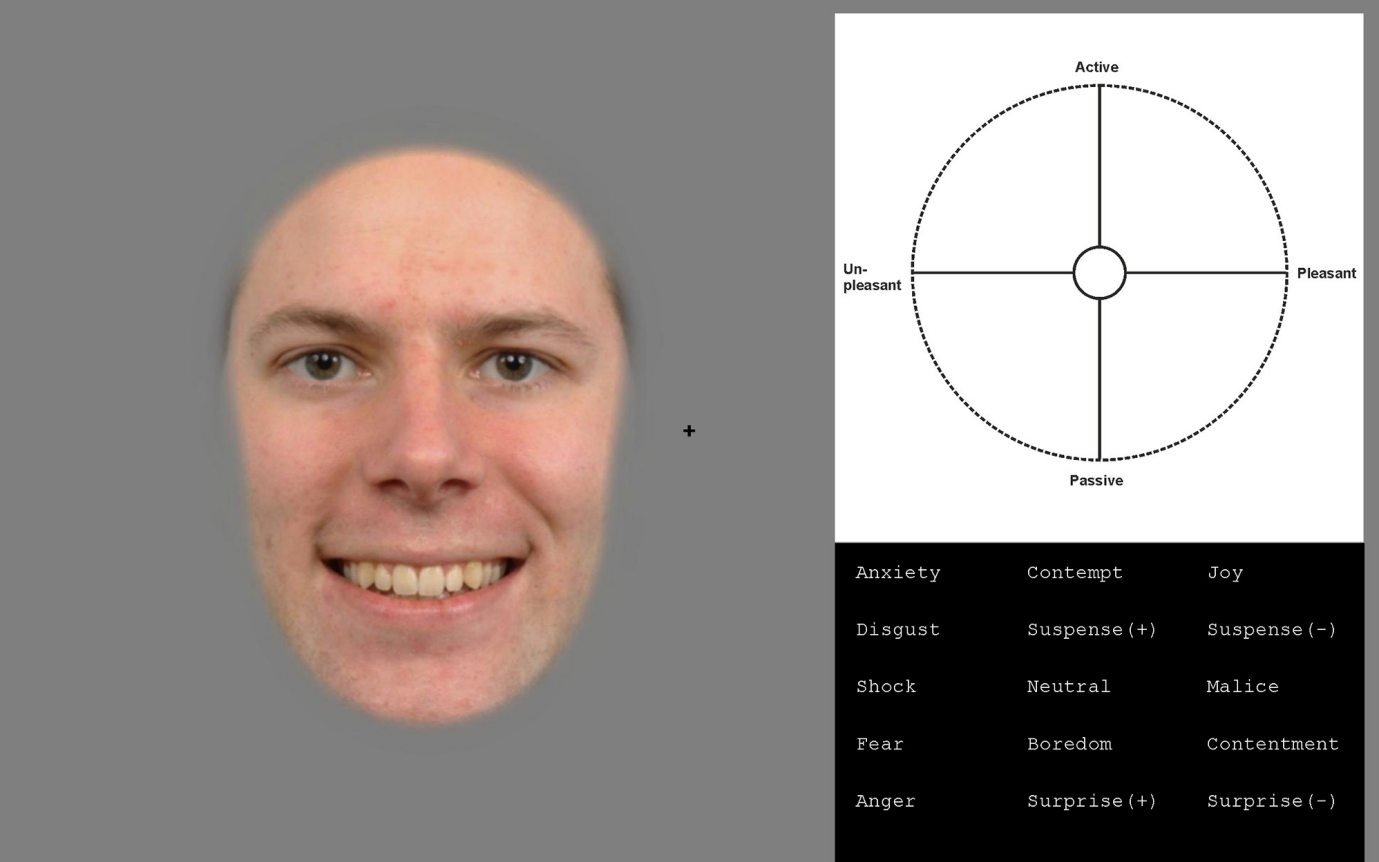
Example of the stimulus display presented to participants at the beginning of a trial. During each trial one facial image was presented on the left side of the screen and the V-A space and the term list (black background) on the right side of the screen. The participants’ task was to first give a rating within the V-A space (top right) and then to select 1–2 terms (bottom right) that best described the emotional expression of the presented face. The participant had up to 3.5 seconds to view the facial image on left, after which it disappeared. The expression portrayed in the example here represents a happy mouth combined with neutral eyes. The example face image is adapted from Radboud faces database (http://www.socsci.ru.nl:8180/RaFD2/RaFD?p=main). The copyright holder, Radboud faces database, has given a written informed consent to publish this image.

In this study, we investigated the perceived expressions of composite faces in an explorative manner. We showed participants composite face stimuli where the expressions of the eyes and the mouth were independently picked from seven alternatives (Neutral, Anger, Disgust, Fear, Happiness, Contempt, and Surprise). The task of the participants was to indicate the perceived affect by clicking any point within the valence-arousal (V-A) -space [16; see Fig 1]. The point-and-click task we used should be relatively neutral in respect to categorical and dimensional theories of emotions and should not bias subjects’ responses in neither direction. To anchor the results of this somewhat abstract method to a more established practice, participants also chose terms from a list of 15 adjectives to describe the expressions. The eye movements of some participants were also tracked in order to determine if different expressions induced different eye movement patterns.

If participants clicked roughly on the same points when rating incongruent (e.g., happy mouth and fearful eyes) and congruent (e.g., happy or fearful) facial expressions, that would support the categorical theory of emotion perception. In contrast, if participants clicked on a point roughly half-way between the points they clicked on when viewing the congruent expressions, such a result would provide support for the dimensional perception theory. While it is interesting to compare our results to the clearly different predictions of the categorical and dimensional theories, we emphasize that our study is considerably more general in scope. The results provide a comprehensive mapping between the emotion signals conveyed by the eye and mouth regions and the perceptions those signals elicit in human observers.

## Materials and methods

### Participants

The participants, 21 women and 11 men (age 20–36, mean 25 years, men about 4.3 years older (t(30) = 3.1, p<0.01)), were all university students, but we made an effort to include students with variable ages and study backgrounds. The sample size was determined based on similar studies published previously [eg., 11]. Participants were asked for difficulties in face perception. However, subjects did not report such problems. Thus, no recruited participants were excluded, for any reason. They received a movie ticket for their participation. The study adhered to the principles of the Declaration of Helsinki and was considered ethically acceptable by the Ethics Review Board in the Humanities and Social and Behavioural Sciences of the University of Helsinki. Participants signed written informed consent, prior to the experiment. Eye tracking data was collected while the participants carried out the expression rating task. Due to technical difficulties (data corruption), eye tracking data could be analysed only for 17/32 participants. However, the distributions of age (t(30 = 0.69, p = 0.5), stimulus sets (χ2(1)<0.35, p>0.55) and sex (χ2(1)<0.22, p>0.63) were very similar in the eye-tracking population (mean age 26 years, SD 4.36; 41% stimulus set 1; 59% women) and in the whole population (mean age 25.1 years, SD 4.26; 50% stimulus set 1; 66% women). The experimental session for each subject lasted about an hour.

### Stimuli

Frontal, full-colour images of the faces of 12 adult Caucasian individuals from the Radboud faces database [17] were used as stimuli. Although the effects of emotional expressions should be highly generalizable across identities [18,19], we wanted to avoid effects due to idiosyncratic characteristics of individual identities and thus used several identities. For each identity, images of seven facial expressions were selected: neutral, anger, disgust, fear, happiness, contempt and surprise. Sadness was not included since we expected sadness to be perceived very similarly to the neutral expression, especially when a sad mouth is combined with another expression in the eyes. Our previous study also suggests that sadness is the least precisely identified, discriminated and remembered expression [20].

When constructing the composites, all images were first spatially scaled and aligned, and then the face was cropped using an oval mask. Then, the upper and lower halves of the face were cropped using spatial Gaussian windows, and all possible combinations of the upper and lower halves of the different expressions were created. Faces were split in half at the mid-nose point and, hence, the expression of the lower part of the nose (nostrils) corresponded to the expression of the mouth, while the expression of the upper part of the nose corresponded to the expression of the eyes. For each identity, 49 different stimulus images were created depicting 7 congruent and 42 incongruent expressions. The height and width of the faces were 12.5 and 8.7° (of visual angle), respectively. Fig 1 provides an example of the stimuli. The location of the face was such that the centre of the face was on average vertically aligned at the level of the fixation cross and horizontally at 5.7° eccentricity. The location varied from trial to trial in both horizontal and vertical directions by ±0.93°.

### Apparatus

Stimuli were created with Matlab 8 (Mathworks, Natick, MA,USA), running on a PC with an Nvidia Quadro K5000 (Nvidia, Santa Clara, CA, USA) graphics card, and presented with the Psychophysics Toolbox 3 [21] on a 22.5” VIEWPixx (VPixx Technologies Inc., Quebec, Canada) display with a 120 Hz refresh rate and background luminance of 104 cd/m^2^. The monitor subtended 29.2° horizontally and 18.5° vertically at a viewing distance of 93 cm. Eye movements were recorded using the Eyelink 1000 (SR Research, Missisauga, Canada) video eye tracker at 1000 Hz, using a chin rest. Both pupil and corneal reflection were used for tracking. The eye tracker was controlled by means of the Eyelink toolbox for Matlab [22]. The standard 9-point calibration procedure was used.

### Procedure

The trial started with the presentation of the fixation cross. After 600 ms the facial image appeared on the left-hand side of the screen, while the V-A space and the term list appeared on the right-hand side of the screen (Fig 1). The facial image was presented for 3500 ms. The V-A space and the term list remained on the screen until the participant provided two separate responses: first, a V-A rating by clicking with a mouse on a point within the V–A space and, and second, a verbal judgement by selecting with a mouse up to two terms in the term list. The terms included on the list relied on previous studies [23] and our unpublished pilot study. We used the following terms: Anxiety, Disgust, Shock, Fear, Anger, Contempt, Joy, Suspense (positive), Suspense (negative), Neutral, Malice, Boredom, Contentment, Surprise (positive), and Surprise (negative). Participants were allowed to select a specific term twice. The gaze was tracked throughout the trial, but only fixations within the face area were analysed. In total, each participant completed 245 trials, consisting of 5 ratings of 49 composite emotional expressions. The different identities were balanced, such that each participant viewed six (three women, three men) different identities. Each identity was used in 40–41 trials per participant.

### Data analysis

The experiment produced two sets of behavioural data, coordinates in the valence-arousal (V-A) space and frequencies of emotional terms. The first data set, the V-A space coordinates were obtained simply from the points in the V-A space (see top right of Fig 1) that the participant clicked while rating each expression. Thus, the data can be directly averaged, analysed and illustrated in the context of the V-A space without any transformations. Each participant’s V-A space rating for a specific expression was produced by averaging the valence (i.e., horizontal) and arousal (i.e., vertical) coordinates of the five click responses the participant gave for that specific expression. These rating coordinates were then used in further analyses. The statistical significance of the differences between the V-A rating coordinates for congruent expressions were analysed with a repeated measures MANOVAs, with the rating coordinates as the dependent measures and the emotional expression as a within subjects factor. The effects of changing the expression of the eye or the mouth region from a congruent expression were analysed in three steps. Firstly, to concentrate on rating shifts with meaningful amplitude, only shifts larger than the SD of the ratings of the congruent expression to which the change was made were included in further data analysis. Secondly, as we were interested in shifts to unexpected directions, we used t-tests to determine the shifts that deviated statistically significantly from the straight line between the rating locations of the two relevant congruent expressions. Thirdly, for the rating shifts that deviated significantly from the expected direction, we used repeated measures MANOVAs (and pairwise post-hoc tests) to test whether the location of the incongruent expression indeed differed significantly from the rating locations of the relevant congruent expressions. Bonferroni correction was applied to the t-tests and the MANOVAs. To avoid violating the normality assumption, one outlier was excluded from MANOVAs involving the angry expression condition (in the congruent angry expression condition, the outlier participants average V-A rating along the arousal dimension was more than 3.8 SDs from the mean across participants, Shapiro-Wilk (32) = 0.87, p<0.01). In addition, two participants’ ratings in the condition with neutral eyes and a disgusted mouth were more than 3.5 SD from the mean in the arousal dimension (Shapiro-Wilk (32) = 0.791, p<0.01). Those cases were thus removed from MANOVAs involving that condition. Additionally, we tested the differences of the ratings of all 49 stimuli. Conventional repeated measures MANOVA cannot be applied to an omnibus test of all (7*7) conditions, as the number of conditions is larger than subjects. Instead, we used two recently developed MANOVA packages for R [24,25,26] that can handle such data. They don’t require normally distributed data, either, and all cases were thus included in that analysis. These MANOVAs rely on ranks and resampling rather than the traditional F-test. Thus, F- and df-values cannot be reported for these tests.

The second set of behavioural data consisted of the terms that participants had selected from a list (see bottom right of Fig 1) to describe the expressions. Term list selections could not be averaged and were thus treated as individual responses. Further, term selections cannot readily be presented in the V-A space. To facilitate comparison of the selected terms and the V-A space ratings, we conducted a multidimensional scaling (MDS) analysis. In the MDS analysis, we first calculated the frequency of all terms across all subjects for the seven congruent expressions. This resulted seven in vectors of term frequencies. Then a dissimilarity matrix was obtained by calculating Pearson correlation of the term frequencies and subtracting the correlation matrix from one. Next, mdscale–function in Matlab was used to project the data to 2-dimensional space. Finally, this MDS map was isotropically scaled and rotated to fit the average V-A space click rating data by minimizing sum of squared differences between the MDS and mean rating coordinates.

In addition to behavioural data, various eye tracking measures were analysed separately using the repeated measures ANOVA, where expression and congruency served as the independent variables.

## Results

**Fig 2:**
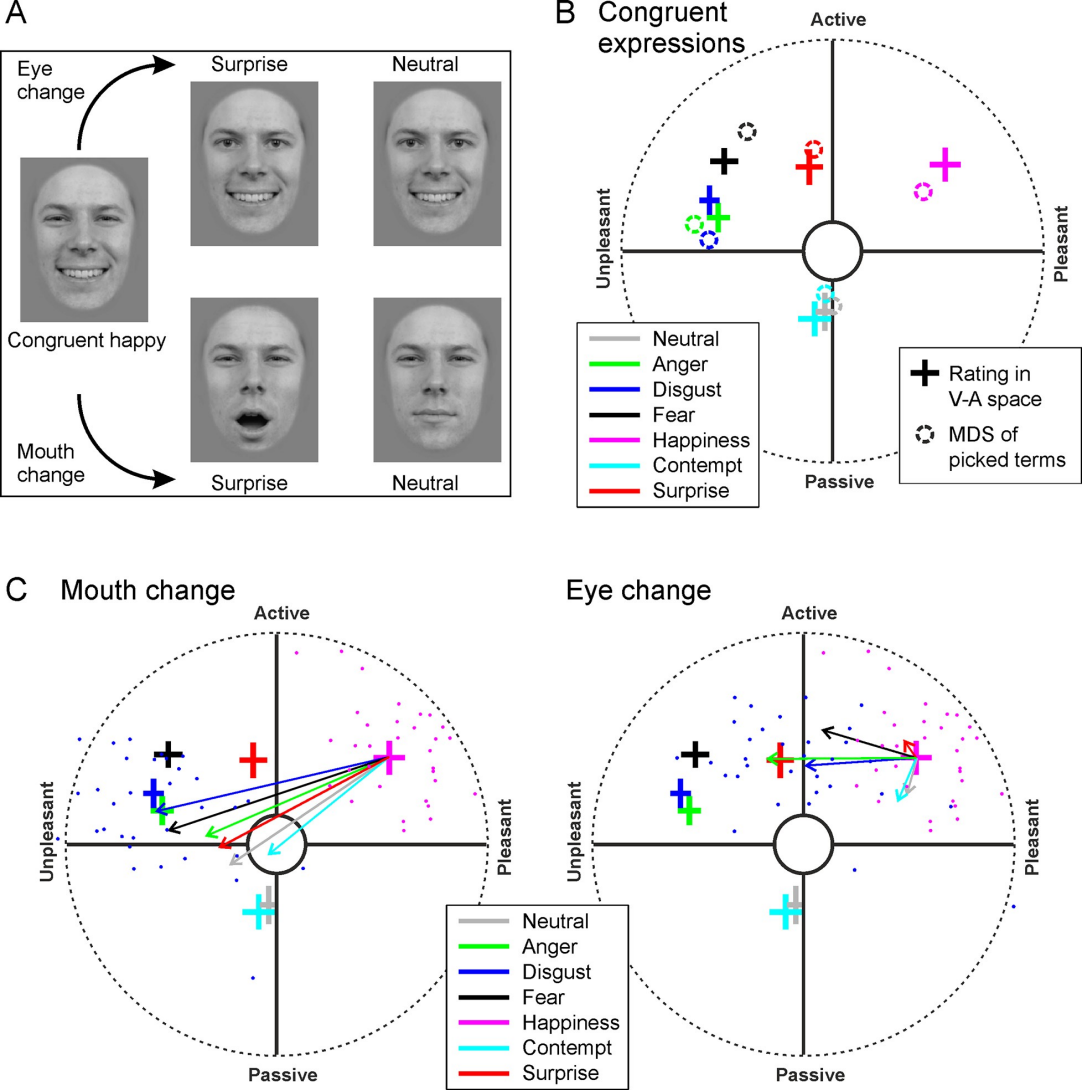
Data from the two measurement methods are in agreement. **A)** Examples of the congruent and incongruent stimuli used in the experiment. **B)** Perceptions of congruent emotional expressions as reflected in the direct V-A-ratings (crosses, where the width indicates the 95% CI over participants) and MDS analysis of terms selected from a list (circles). For presentation purposes, the MDS analysis result was transformed, whilst keeping the structure of the data intact (isotropically scaled and rotated). Our simulations (10 000 randomizations) showed that the probability that such a strong correspondence (as indicated by sum of squares of location differences) between the structures of the rating data and the MDS analysis would occur by chance is very low (p<0.001). **C)** Examples of changes in the perception when either the mouth (left) or eye (right) region was changed from the congruent expression. The point of the arrow indicates the point to which perception on average shifted in the V-A space. For example, when mouth was changed from happy to disgusted (left), whilst the eyes remained happy, perception shifted quite completely to a point corresponding to the perception of congruent disgust (long blue arrow originating from the magenta cross). Each small dot represents the average of one participant’s ratings in corresponding conditions (magenta: congruent happy, blue: mouth (left) or eyes (right) changed to disgusted).

In general, both the eyes and the mouth had clear effects on the perceived emotional expression, and there were also pronounced interaction effects (see Fig 2C, for examples). In a repeated measures MANOVA with Mouth expression (7 categories) and Eye expression (7 categories) as factors and the valence and arousal click coordinates as dependent variables, both main effects and the interaction effect were statistically significant (p<0.001, see Data analysis for details).

In the following, we will first present data on how the participants perceived the congruent emotional expressions. After that, we will consider how various incongruent expressions relate to the congruent expressions they are built from.

### Perceptions of congruent expressions

The average valence-arousal (V-A)–ratings (which subjects gave by clicking a point in the V-A space, see top right of Fig 1) for the congruent stimuli were in striking agreement with the multidimensional scaling (MDS) analysis of the terms that participants used (by picking terms from a list, see bottom right of Fig 1) to describe the expressions (Fig 2B). In the MDS analysis, a dissimilarity matrix (1 –correlation of term frequencies across all participants) was projected to a two-dimensional space (see methods for more details). The high overlap, which is very unlikely to occur by chance (see legend of Fig 2), suggests that the participants correctly understood the V-A space and the task, and were quite systematic in their responses. In general, the relative V-A locations of the congruent facial expressions, that were found in this study, are in good general agreement with previous studies, where participants separately rated the valence and activation [27,28].

Somewhat surprisingly, participants placed contempt in the same V-A location with neutral expressions: slightly passive in arousal, but rather neutral in the valence dimension (Fig 2B). In fact, in a repeated measures MANOVA using the valence and arousal coordinates of participant’s V-A space clicks as the dependent variables and the congruent expression as the independent variable, neutral and contempt represented the only expressions which did not differ statistically significantly(*Wilk’s Λ* = 0.92, *F*(2,29) = 1.3, p = 0.28, $\eta_{p}^{2}=0.084$). Although the difference between the ratings of angry and disgusted expressions is modest (Fig 2B), it is statistically significant (*Wilk’s Λ* = 0.75, *F*(2,29) = 5.7, p = 0.014, $\eta_{p}^{2}=0.254$).

In addition, contempt emerged as quite similar to the neutral expression when the selected terms were examined. Both were often considered boring and neutral. For the neutral expression, however, many rated the expressions as ‘not recognised’ within the V–A rating response. For the contemptuous expression, the ‘not recognised’ rating was rarely used. Instead, the contemptuous expression was mostly perceived as reflecting boredom (84% of trials) or neutral (34%), but contemptuous quite rarely (21%). The sum of percentages is over 100, as participants could choose up to two terms.

### Perceptions of incongruent expressions

The differences between the 49 rating averages and, moreover, the relationships between congruent and incongruent expressions, are difficult to grasp if all the ratings are simultaneously illustrated in the V-A space. Therefore, to guide the reader through our results, we first provide an example of how the incongruent expressions were perceived in comparison to the congruent expressions and then present the entire dataset in a more compact form. Further, all possible unique pairwise comparisons (49*24 = 1176) between different expressions are beyond the scope of this article. For the interested reader, however, we provide the fdr corrected p-values and the effect sizes of all pairwise comparisons, as well as rating averages plotted in the V-A space as supplementary Figures (S1 Fig). Out of 1176 tests, only 46 were not statistically significant, indicating that most of our stimuli provoked quite unique and consistent perceptions.

The composite faces were presented in a completely randomised order, and the participants were not tasked with comparing different expressions. Nevertheless, since were are mainly concerned with how the rating of each incongruent expression (e.g., happy mouth, angry eyes) relates to the ratings of the relevant congruent expressions (i.e., congruent happiness and congruent anger), it is useful to consider the various incongruent expressions as changes from congruent expressions. In Fig 2C, the arrows indicate how much and in which direction the average rating shifted when the mouth (left) or eyes (right) changed from the congruent happy to another expression. The colour of the arrows indicates the emotion to which the expression of mouth (left panel) or eyes (right panel) changed (see legend), whilst the arrow tip indicates the V–A space location to which the ratings on average shifted. Keeping the eyes happy and changing the mouth (Fig 2C, left) carried a much stronger effect than keeping the mouth happy and changing the eyes (Fig 2C, right). The ratings were consistent across participants, as shown by the small confidence intervals (Fig 2, size of the crosses) and the agreement across ratings from different participants (Fig 2C, small blue and magenta dots).

**Fig 3:**
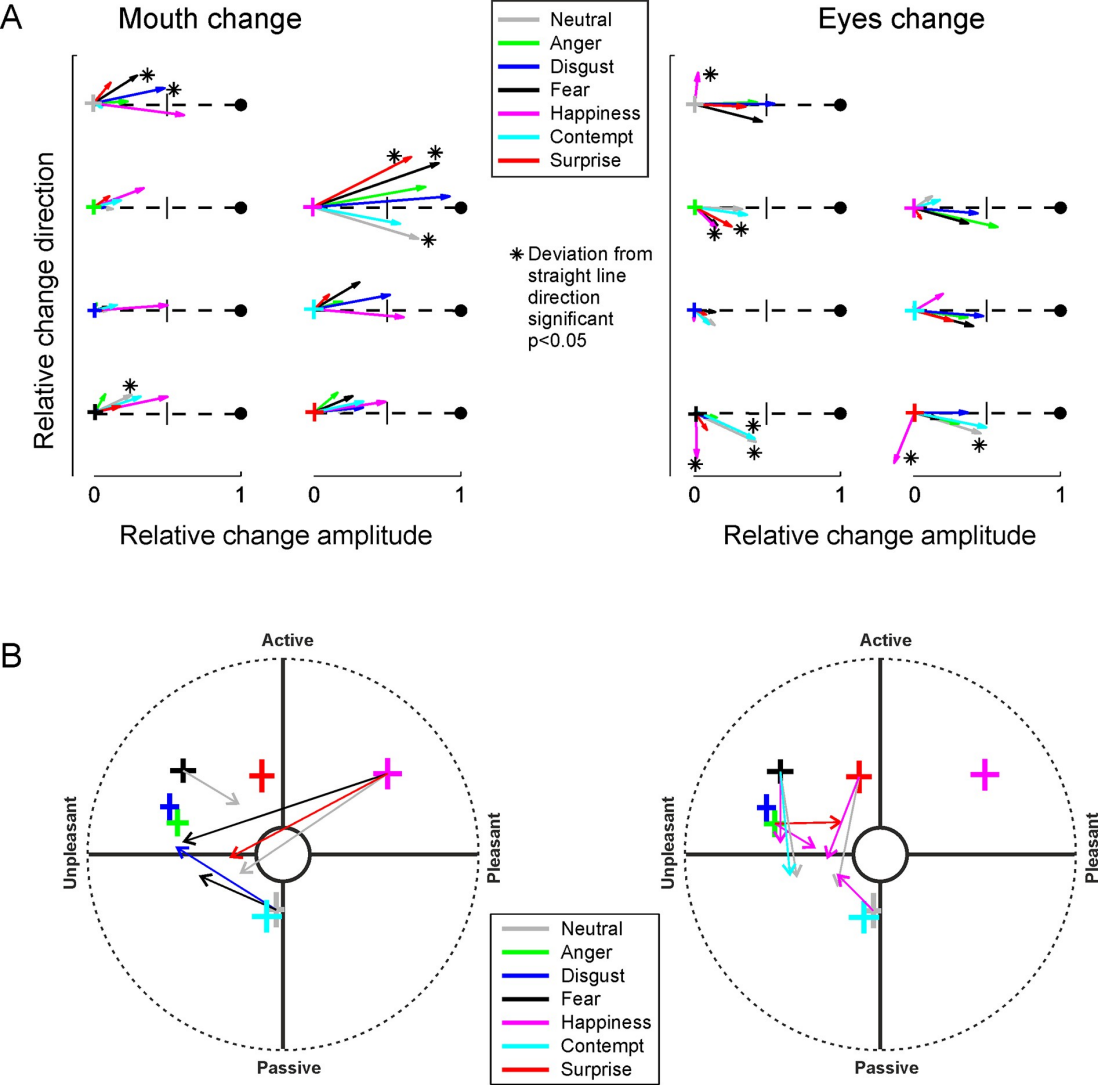
Shifts in perceived emotions due to incongruence. **A)** All shifts caused by changing the expression in the mouth region (left) or the eye region (right). The widths of the crosses indicate the 95% CIs (across participants) for the ratings of congruent expressions (see labels), the colours of the crosses indicate the congruent expression from which the mouth or eyes were changed. The arrows represent the perception shift caused by changing the eye region (left) or the mouth region (right) to that indicated by the arrow colour (see legend). The length of the arrows represents the relative magnitude of the change. An arrow that reaches the vicinity of the black circle at the end of the dashed line indicates that the percept completely followed the changed feature, an arrow that reaches the line perpendicular to the direct (dashed) path towards the centre indicates a percept based equally on both regions. Asterisks indicate shifts to directions which were of considerable magnitude but towards a direction that differs statistically significantly (t(31)>2.9, p<0.05 with Bonferroni correction in all tests, see Tables 1 and 2) from the direction expected based on the two emotions present in the composite faces (the dashed line). **B)** The unexpected shifts (indicated with asterisks in A), now presented in the V-A space.

Fig 3A illustrates all of the perceptual shifts caused by changing the expression of the mouth region (left) or the eye region (right) from a congruent expression. One can see that changes in mouth and eyes carry quite different effects. Firstly, changing the mouth region shifted the ratings more, on average, than a change in the eye region. This difference is illustrated by the longer arrows in the left panel than those in the right panel. The difference was statistically significant (t(31) = 4.867, p<0.001, $\eta_{p}^{2}=0.43$). The means (and SDs) of the relative change amplitude (length of arrows) between the expressions reached 0.42 (0.086) for changing the mouth and 0.36 (0.093) for changing the eyes. We must note, though, that this difference is largely caused by the dominance of the mouth in the happy expression. When the mouth was changed from a congruent happy mouth to any another expression, the perception strongly shifted, almost directly towards the other expression (arrows from the magenta cross in Fig 3A, left). Other congruent expressions were similar in that changing the mouth expression to happy shifted the percept towards happy quite strongly.

**Table 1:** Statistical test results of the significant deviations (see asterisks in Fig 3A) caused by changing mouth expression from a congruent expression. Bonferroni correction has been applied to all p-values. In MANOVAs df = 4,26 in the condition marked with *, df = 4,28 in all others. All pairwise differences (post-hoc tests in MANOVAs) are statistically significant (p < 0.001).

Congruent	Mouth	Angular deviance		MANOVA			
origin	changed to	t	p-value	*Wilk’s Λ*	F	p	$\eta_{p}^{2}$
Neutral*	Disgust	4.30	0.007	0.077	78.4	<0.001	0.923
Neutral	Fear	5.27	<0.001	0.078	83.1	<0.001	0.922
Fear	Neutral	3.72	0.033	0.074	87.1	<0.001	0.926
Happiness	Neutral	5.03	<0.001	0.057	116	<0.001	0.943
Happiness	Fear	6.36	<0.001	0.055	121	<0.001	0.945
Happiness	Surprise	5.27	<0.001	0.050	133	<0.001	0.950

**Table 2:** Statistical test results of the significant deviations (see asterisks in Fig 3B) caused by changing eye expression from a congruent expression. Bonferroni correction has been applied to all p-values. In MANOVAs, df = 4,27 in conditions marked with *, df = 4,28 in all others. All pairwise differences (post-hoc tests in MANOVAs) are statistically significant (p < 0.001).

Congruent	Eyes	Angular deviance		MANOVA			
origin	changed to	t	p-value	*Wilk’s Λ*	F	p	$\eta_{p}^{2}$
Neutral	Happiness	4.34	0.006	0.057	116	<0.001	0.943
Anger*	Happiness	4.88	0.001	0.046	141	<0.001	0.954
Anger*	Surprise	4.54	0.003	0.154	37.1	<0.001	0.846
Fear	Neutral	5.27	<0.001	0.078	83.1	<0.001	0.922
Fear	Happiness	18.07	<0.001	0.055	121	<0.001	0.945
Fear	Contempt	5.27	<0.001	0.088	73.0	<0.001	0.912
Surprise	Neutral	3.73	0.033	0.131	46.4	<0.001	0.869
Surprise	Happiness	4.34	<0.001	0.050	133	<0.001	0.950

Our rating method revealed shifts in an unexpected direction. That is, we identified cases where the rating of an incongruent expression did not, even approximately, lie in the area between the ratings of the two congruent expressions that the composite was made of. Such unexpected ratings, for which the deviation was statistically significant (see caption for Fig 3 for further information and Tables 1 and 2 for statistics) are indicated by asterisks in Fig 3A, and replotted in V-A space in Fig 3B. The most consistent and surprising finding is that when the eyes changed to the happy expression from multiple other congruent expressions (fear, anger, neutral and surprise), the ratings (the magenta arrows in the right panel) did not shift towards the rating of the congruent happy expression, but towards a common point slightly negative along the valence dimension and neutral along the arousal dimension. Accordingly, participants did not select ‘joy’ from the term list when viewing a face that combined happy eyes with a neutral or surprised mouth. Indeed, as the examples in Fig 2A show, happy eyes combined with a fearful or neutral mouth do not appear particularly joyful.

### Eye tracking

Regarding eye movement behaviour, the stimulus conditions mainly affected the total dwell time, i.e., the total time spent fixating any part of the facial image (including re-fixations after intervening fixations on the V-A space). The total dwell time was significantly affected both by congruency (F(1,16) = 8.809, p = 0.009, $\eta_{p}^{2}=0.36$) and expression (F(6,96) = 2.671, p = 0.019, $\eta_{p}^{2}=0.14$). The interaction effect was also significant (F(6,96) = 3.202, p = 0.007, $\eta_{p}^{2}=0.17$). Mean (and SD) over participants was 1967 ms (565 ms) in the congruent condition and 2051 ms (479 ms) in the incongruent condition. Congruency had a significant effect also on the latency to first fixation on the face (F(1,16) = 5.430, p = 0.033, $\eta_{p}^{2}=0.25$), but there was no effect of expression (F(3.3,52.8) = 1.947, p = 0.128) or interaction effect F(2.77,44.3) = 0.763, p = 0.511). Mean (and SD) over participants was 189 ms (20 ms) in the congruent condition and 204 ms (29 ms) in the incongruent condition.

For the location of the initial fixation on a face, we found no significant effect along the horizontal location (congruence: F(1,16) = 0.556, p = 0.467; expression: F(6,96) = 0.993, p = 0.435; interaction: F(6,96) = 0.705, p = 0.646), whilst the effect of the expression was significant along the vertical location (F(6,96) = 2.259, p = 0.044, $\eta_{p}^{2}=0.12$). The effect of congruence (F(1,16) = 0.001, p = 0.995) or the interaction effect (F(6,96) = 0.701, p = 0.649) were not significant. The only significant difference in pairwise comparisons was that between congruent disgust and fear (t(16) = 3.138, p = 0.006, $\eta_{p}^{2}=0.38$). On average, the initial fixation on a face expressing disgust was placed about 0.27° lower than on a face expressing fear.

## Discussion

We studied how people perceive emotional expressions on congruent and incongruent composite faces. The task of the current study was somewhat abstract, since participants provided their ratings by directly clicking on a point within the valence-arousal (V-A) space, which represents a theoretical description of the structure of emotions. Nevertheless, the overall structure of the ratings of congruent expressions closely agreed with the structure observed in earlier studies with various methods [reviewed in, 29]. More specifically, happiness is alone in the high valence, high arousal quadrant, whereas fear, anger and disgust are near each other on the unpleasant side with fear falling somewhat higher along the arousal dimension than anger and disgust. In particular, Gerber et al. [30] used a very similar task of estimating the expression in the V-A space and found a structure that mirrors ours.

After rating the facial expressions within the V–A space, participants also selected two terms (from a 15-term list) that best described the facial expression. As a result, we were also able to compare the V-A space ratings to the structure provided multidimensional scaling applied to the term selection frequencies. The structures of the two datasets were in rather remarkable agreement. Since participants always selected the terms after providing the V–A rating for each trial, some similarities were expected merely on the basis of participants consistently selecting the same terms after selecting a particular location within the V–A space. However, this does not explain the similarity in the relative distances between the congruent expressions in the two datasets. The agreement between the two tasks provides further evidence that our V–A space task served as a valid measurement of perceived emotion. Consistent with [11], this suggest that the task does not determine results and that emotions can be similarly evaluated by selecting specific terms or by clicking points in V-A space.

When participants viewed incongruent expressions, the ratings of the expression were generally more strongly affected by the mouth than the eyes, although anger emerged as a notable exception (see Fig 3A). This is consistent with the mouth region’s higher salience as an information source [4,31]. In the current study, this dominance of the mouth region was most pronounced for the expression of happiness (see the magenta arrows in Fig 3A, left), perhaps reflecting the tendency of a smiling mouth to directly affect the interpretation of eye expressions [32].

By contrast, happy eyes usually affected the percept rather weakly, often to an unexpected direction. When the expression of eyes was changed to happy from another congruent expression, the rating in many cases shifted towards a point in the V-A space that was slightly negative in valence and neutral in arousal, rather than towards the positive, high arousal quadrant occupied by the congruent expression of happiness (Fig 3B, right). Indeed, Fig 2A illustrates how these composite expressions appear quite negative. Although eyes expressing joy are widely considered instrumental to the expression of genuine joy [33] and happy eyes shown separately do signal joy rather effectively [34], it has earlier been observed that eyes provide a weak cue for the discrimination between expressions of happiness and sadness [35]. Our results suggest that happy eyes not only provide a weak signal, but may often be perceived as a slightly negative signal when combined with other mouth expressions.

Neither the categorical nor the dimensional theory of emotion perception is unequivocally supported by our results. In some cases the perceived emotion was completely unaffected by changing the expression in the eyes or the mouth. Yet, in other cases the perception reflected a relatively evenly weighted integration of the two combined expressions (see e.g., the blue arrow and points in Fig 2C, right). Fujimura et al. [11] presented morphed face stimuli that varied continuously between two expressions (e.g., from happiness to fear). They found that perception sometimes followed the stimulus change in a continuous, dimensional manner, but at times strictly adhered to distinct categories. Our results agree with those of Fujimura et al. [11], thus supporting a hybrid theory of emotional expression [13]. It has been suggested that the evaluation of emotions constantly interacts with cognition and the evaluation of mental processes [13]. Some of our incongruent stimuli might be perceived not purely emotional, but containing a combination of emotion and a mental state. For example, happy mouth combined with angry eyes might be perceived as reflecting mental states such as ashamed or baffled. Indeed, a variety of expressions [36] and complex mental states [15], which partly overlap in V-A space, can be facially expressed and recognized. The large variability of facial expressions is difficult to explain with only a few emotional categories or dimensions.

Interestingly, the rating for the congruent expression of contempt completely overlapped the rating for the congruent neutral expression. Moreover, participants selected boredom from a term list four times as often as contempt when describing a stimulus expressing contempt. Contempt has been the strongest candidate to be added on the list of basic emotions with a unique expression (the unilateral raised lip), but has remained controversial and appears not to be as recognizable as expressions of the “traditional” basic emotions [reviewed in, 37]. In the present study, as in some earlier ones [38], contempt was quite difficult to recognise.

The eye movement patterns were fairly similar with different stimulus types. The most pronounced effect emerged for a longer dwelling time for incongruent face stimuli. This is slightly surprising as the participants had up to 3500 ms to view the face, which was previously found to suffice even in self-paced viewing [39]. The difference in viewing time (about 100 ms) was of a similar magnitude with reaction times in earlier studies where much faster responding was encouraged [8,40]. This suggests that the processing speed difference begins at an early phase of the expression estimation. Indeed, a significant albeit much smaller (15 ms) difference emerged in the latencies of first fixation. It appears that either the participants noticed the incongruence and used more time to interpret the emotion in the face or integrating inconsistent cues requires more processing time.

The somewhat modest sample size, the statistically different age of female and male participants and relying on the participants’ report on normal face processing abilities are some of the current study’s limitations, which future studies should try to avoid.

In conclusion, we found that although the mouth region generally plays a somewhat stronger role in determining the perceived facial expression of emotion, the relative roles of the eyes and mouth regions depend strongly on the expressions involved. The mouth region rather significantly dominates the perception of a happy expression. Happy eyes, in contrast, rarely represent a positive signal when combined with a mouth that expresses a different emotion. Whether a perceived expression reflects a combination of expressions communicated via the eye and mouth regions or is determined by only one of the regions also depends upon the expression. Future studies using composite stimuli should find our results useful when estimating what sort of perceptions various different stimuli are likely elicit. Also, the rating task combined with incongruent facial images containing happy eyes or a happy mouth could also be utilized when estimating deficits in emotion processing, for example in autism spectrum disorders.

## Supporting information

S1 Fig (DOCX)
