# Supplementary material for: Perceived emotional expressions of composite faces
Source: PLoS One. 2020 Mar 10;15(3):e0230039. doi: 10.1371/journal.pone.0230039 (PMC7064203; doi:10.1371/journal.pone.0230039)
Supplement: S1 Fig — (DOCX) [file pone.0230039.s001.docx]

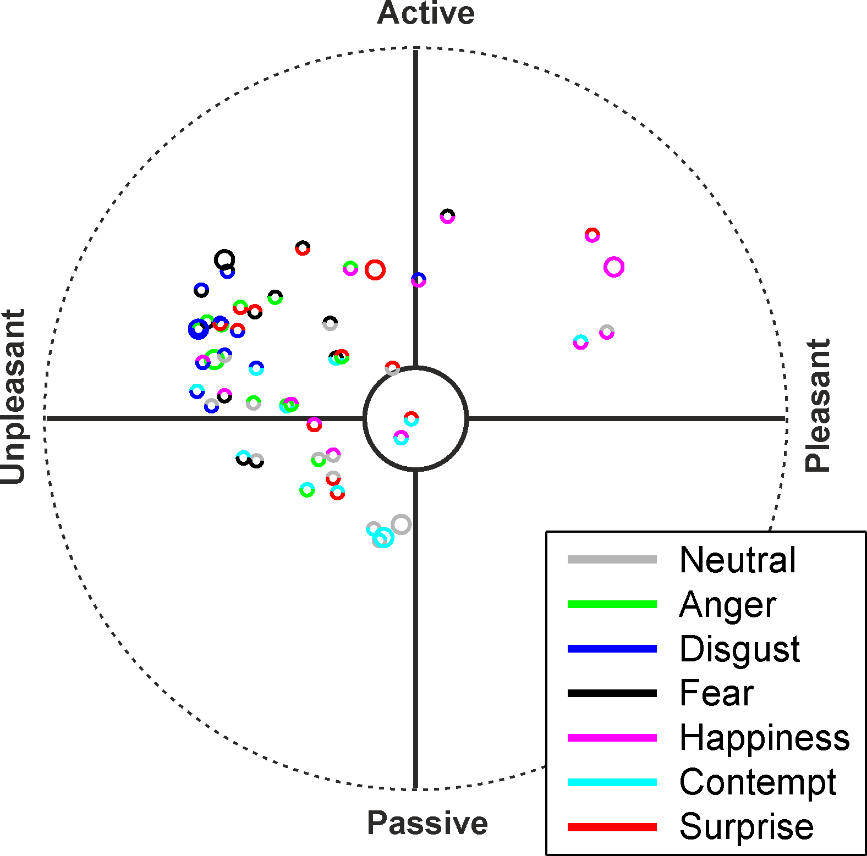


**S1 Fig 1.** Mean ratings of all 49 expressions plotted in the V-A space. The color of the top half of the circle refers to eyes expression, the color of the bottom half to mouth expression (see legend). Single color circles represent congruent expressions.


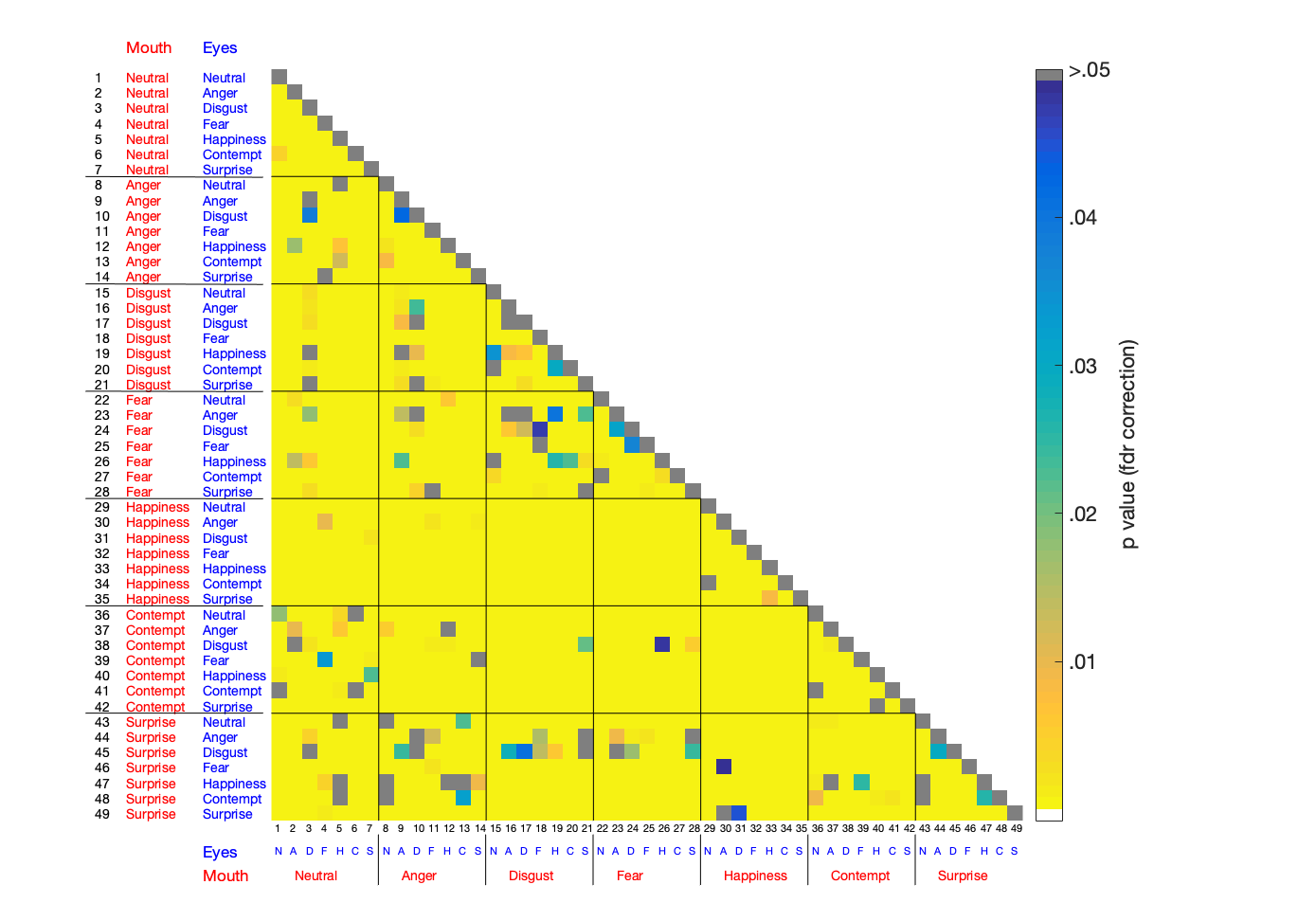


**S1 Fig 2.** P-values (fdr corrected) of all pairwise tests (Hotelling's paired T^2^ -test) of mean ratings of the 49 different expressions, ordered primarily by **mouth** expression. Only 46 tests (gray squares) out of 1176 tests were not statistically significant. N=neutral, A=Anger, D=Disgust, F=Fear, H=Happiness, C=Contempt, S=Surprise. The Matrix in numeric format is available at <https://osf.io/2rqcm/>


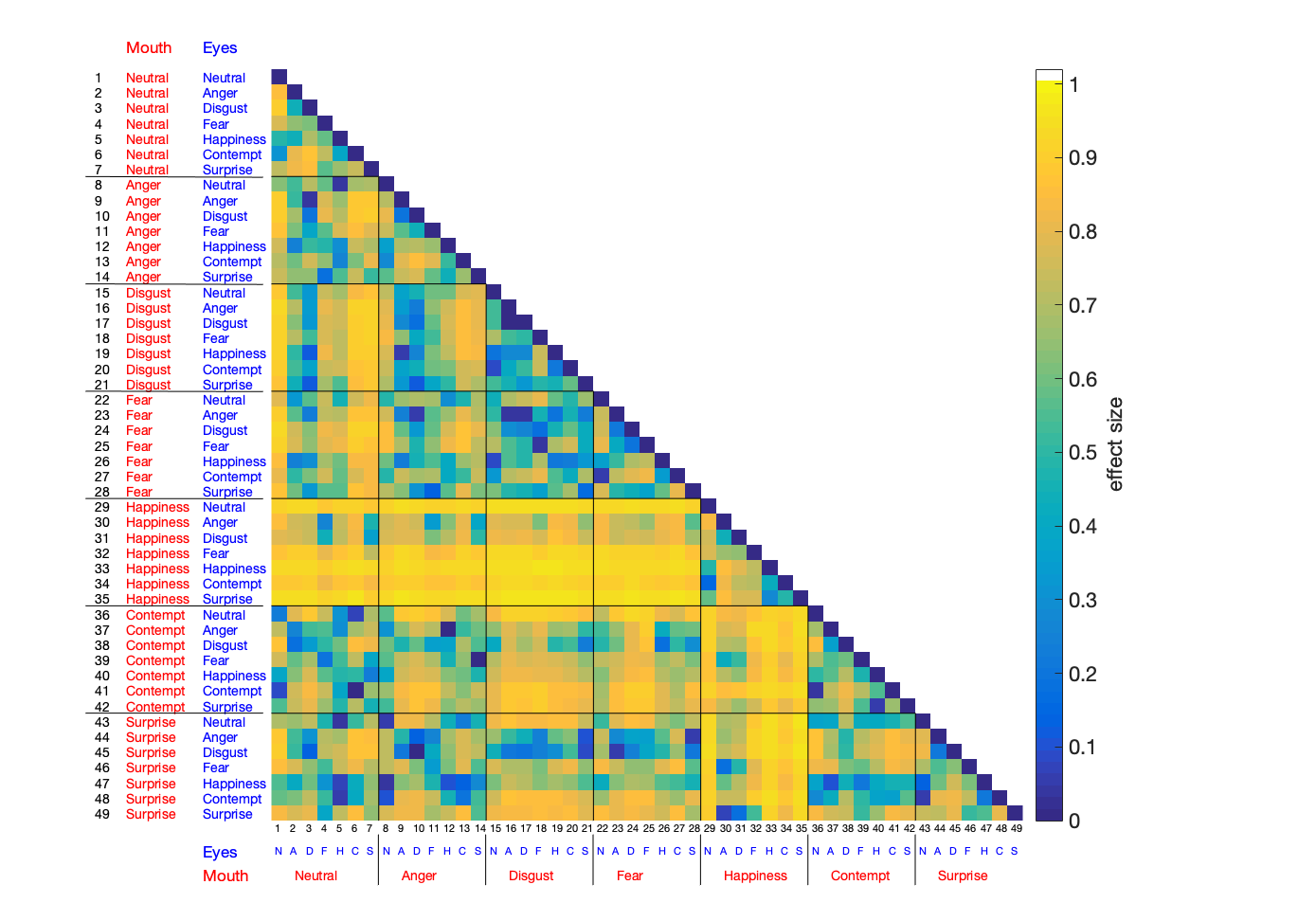


**S1 Fig 3.** *Effect sizes (*$\boldsymbol{\eta}_{\boldsymbol{p}}^{\boldsymbol{2}}$*) of all pairwise differences of mean ratings of the 49 different expressions, ordered primarily by* ***mouth*** *expression. This effect size matrix can be viewed as a dissimilarity matrix: the more yellow the squares, the more different the expression ratings between the stimuli were. For example, faces that contained happy expression in the mouth, were dissimilar with most of other faces.* The Matrix in numeric format is available at <https://osf.io/2rqcm/>


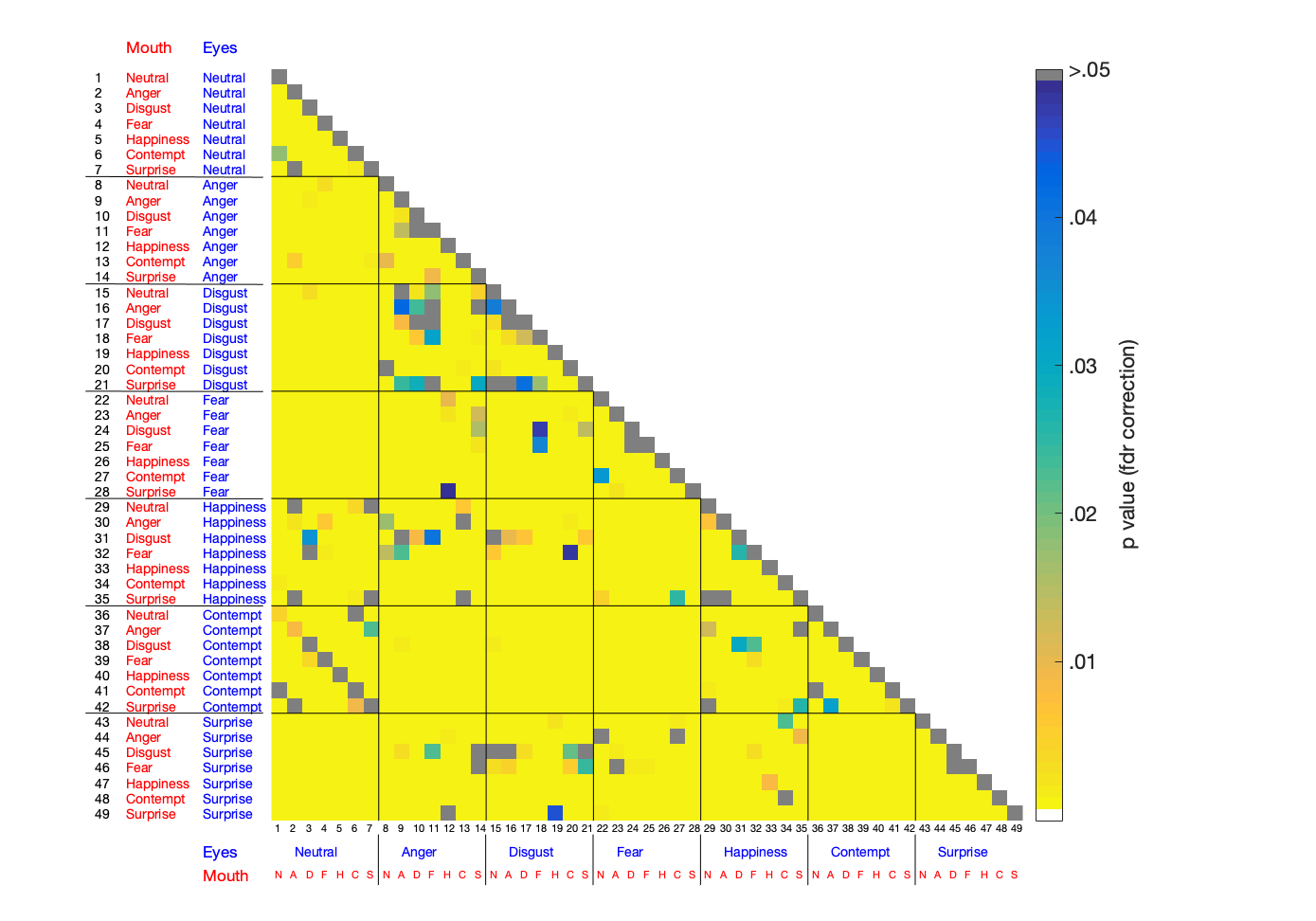


**S1 Fig 4.** P-values (fdr corrected) of all pairwise tests (Hotelling's paired T^2^ -test) of mean ratings of the 49 different expressions, ordered primarily by Expression of **eyes**. Same data as in S1 Fig 2, just re-arranged. The Matrix in numeric format is available at <https://osf.io/2rqcm/>


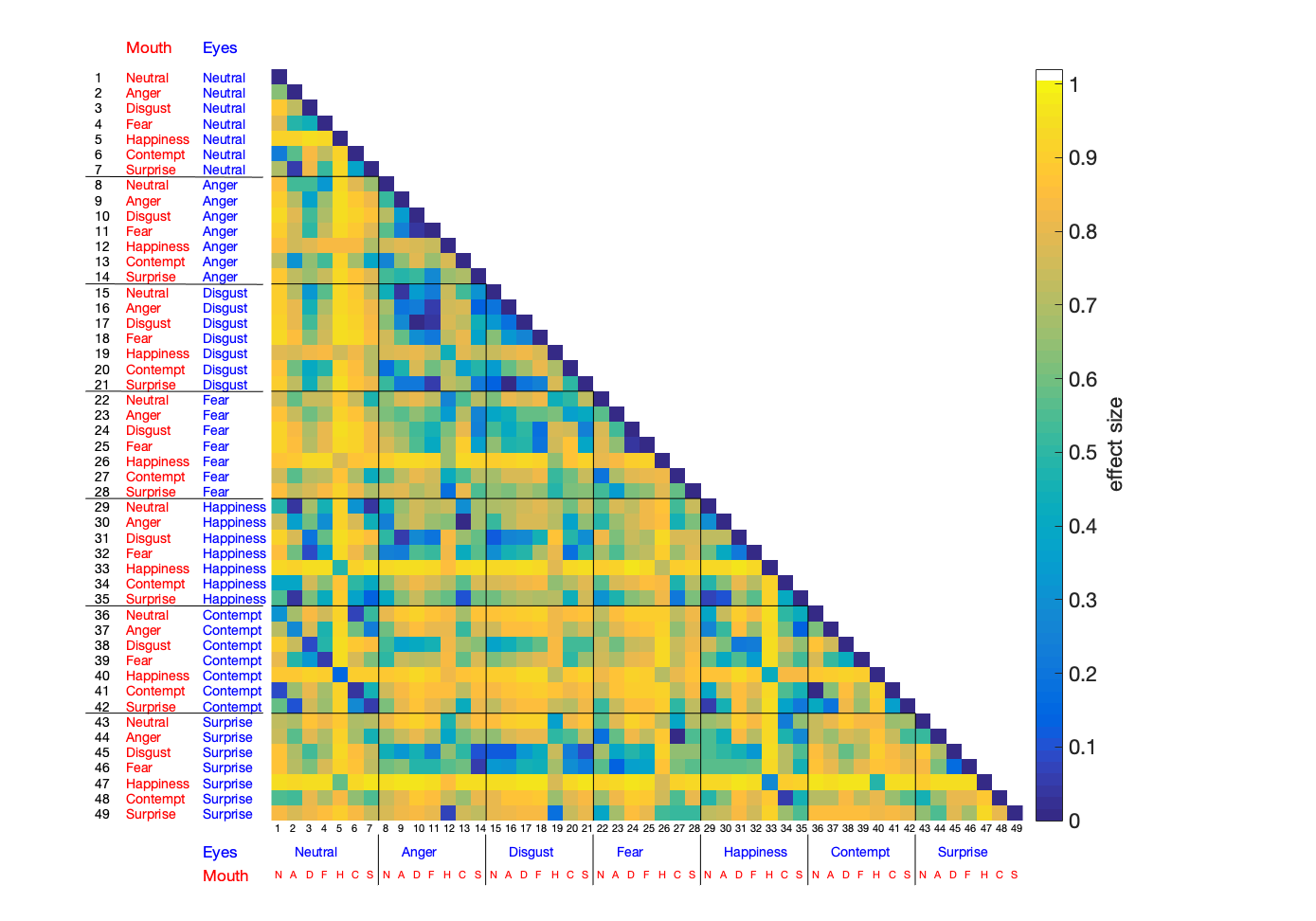


**S1 Fig 5.** Effect sizes ($\boldsymbol{\eta}_{\mathbf{p}}^{\mathbf{2}}$) of all pairwise differences of mean ratings of the 49 different expressions, ordered primarily by **eyes** expression. Same data as in S1 Fig 3, just re-arranged. The Matrix in numeric format is available at <https://osf.io/2rqcm/>
